# **The use of high-frequency ventilation during general anaesthesia: an update**

**DOI:** 10.12688/f1000research.10823.1

**Published:** 2017-05-30

**Authors:** Karolina Galmén, Piotr Harbut, Jacob Freedman, Jan G. Jakobsson

**Affiliations:** 1 Department of Anaesthesia & Intensive Care, Institution for Clinical Science, Karolinska Institutet, Danderyd University Hospital, Stockholm, Sweden; 2 Department of Surgery, Institution for Clinical Sciences, Karolinska Institutet, Danderyd University Hospital, Stockholm, Sweden

**Keywords:** high frequency ventilation, high-frequency jet ventilation, high-frequency positive pressure ventilation

## Abstract

Various forms of high-frequency ventilation (HFV) have been described. HFV is broadly defined as artificial ventilation of the lungs with sub-deadspace tidal volumes delivered using supra-physiological frequencies. HFV has been used in anaesthesia and intensive care for special procedures and conditions since the 1960s. Clinical interest in the use and the technical evolution of HFV has developed over time. There is a renewed interest in HFV for avoiding parenchymal movement during stereotactic tumour ablation. The present paper aims to give an overview of the fundamental physiology, technical aspects, and clinical challenges of HFV in ablation procedures during general anaesthesia, where HFV is used to minimise the movements of the ablation target.

## The development of the different techniques in high-frequency ventilation

High-frequency ventilation (HFV) is an umbrella term covering different techniques including various forms of artificial ventilation that use supraphysiological ventilation frequencies and low volumes (approximating or even less than the anatomical deadspace volume) ^1^. HFV, as it is used today, was invented by coincidence rather than design, as is common with many other inventions. HFV has developed towards three main but somewhat different techniques since it was first invented; high-frequency positive pressure ventilation (HFPPV), high-frequency jet ventilation (HFJV), and high-frequency oscillation ventilation (HFOV). Whether HFOV should be seen as a part of HFV can be argued, but we have included it in the present overview.

HFPPV was described by Öberg and Sjöstrand in 1967 ^2^. HFPPV aimed to minimise the impact of the ventilator on cardiovascular variables (e.g. mean arterial pressure and cardiac output) by keeping peak airway pressure (PAWP) low. Low PAWPs were achieved by delivering low tidal volumes at high breathing frequencies using a specially designed ventilator. After extensive research in animal models, Öberg and Sjöstrand translated the ventilator approach and their newfound knowledge to the operating theatre: HFPPV was used in situations where the surgeon and anaesthetist competed for access to the same anatomical area, such as in laryngoscopy and bronchoscopy, but also in thoracic surgery where minimising lung movement improved surgical conditions. HFPPV gained little popularity outside of Scandinavia, possibly because of the complexity of the technique and the lack of appropriately configured commercially available ventilators ^3, 4^.

The development of HFOV was pioneered by Lunkenheimer and colleagues; they studied myocardial impedance and discovered removal of a significant amount of CO _2 _after oscillation was applied to the airway. In parallel with this work, Bryan and Laws were studying lung impedance during anaesthesia in the early 1970s ^3^. They used a loudspeaker to deliver high-frequency oscillations for measurements of lung impedance and found higher levels of exhaled CO _2_ for each beat. Developing the technique further, they built a high-frequency sine wave generator using a piston pump driven by an electric motor. This arrangement provided excellent gaseous exchange in anaesthetised dogs ^3, 5^. Frequencies of about 900 oscillations per minute were used, as this frequency was thought to be the resonant frequency of the lung. Dogs ventilated with conventional ventilation had lungs that were recruited but de-recruited and became atelectatic during anaesthesia, and the dogs then became hypoxic. In contrast, dogs ventilated with HFOV maintained open lungs owing to the maintenance of a high mean airway pressure (MAWP). This study contributed to the development of the “open lung strategy”, a ventilation concept still used in patients with acute respiratory distress syndrome (ARDS). The open lung strategy aims to prevent ventilator-induced lung injury (VILI).

HFJV was described by Klain and Smith in 1977: “in essence, high frequency jet ventilation (HFJV) consists of the intermittent delivery of gas from a high pressure source through a small-bore cannula positioned in the airway, followed by passive expiration” ^4^. There are a number of different devices for HFJV (see below). The physical arrangements of jets vary considerably depending on the procedure or the situation. The jet cannula is positioned freely in the supraglottic or subglottic region of the airway, connected directly to a laryngoscope/bronchoscope, placed freely in the endotracheal tube, or inserted into the airway percutaneously.

In the adult population, the different HFV techniques may be categorised by frequencies, as suggested by R.B. Smith ^6^. In adults, HFPPV uses frequencies that range from 1–1.8 Hz (60–110/minute), whereas HFJV refers to rates of 1.8–6.7 Hz (110–400/minute) and HFOV refers to rates of 6.7–40 Hz (400 up to 2,400/minute). Another way to describe the difference is to sort these ventilator modalities by the technology being used. This approach is a more appropriate way to discriminate the different HFV modes from each other. HFPPV inflates the airway with fresh gas within a 1–1.8 Hz frequency range, but whether HFPPV incorporates true gas entrainment or not is questioned. HFJV uses a fresh gas jet that simultaneously entrains a second gas. Both HFPPV and HFJV rely on passive exhalation. In contrast, HFOV oscillates the gas back and forth to effect active inspiratory and expiratory phases. Neither the frequency- nor the mechanism-based classification models describe a sudden switch from classical to entirely different physiological methods of ventilation. A further way to define these three ventilatory modalities was suggested by Froese and Bryan in 1987 ^4^. In the same manner as we have two main systems for conventional ventilation, volume control, and pressure control, they propose a system built upon whether expiration is passive or active and add -P or -A after the name of the ventilation: for example, HFJV-P (HFJV with passive expiration). This approach to classification has not been implemented widely, although it might explain differences in outcome.

## Physiology during high-frequency ventilation

In their classic study on panting dogs from 1915, Henderson *et al*. concluded that “there may easily be gaseous exchange sufficient to support life even when tidal volume is considerably less than dead space” ^7, 8^. The physiology behind the gas exchange during HFV is not well defined. There are several theories ^9^, but the exact mode of action is unknown. Six contributing mechanisms are suggested: 1) Taylor dispersion, which is the diffusion of the high-velocity central gases to the margins of the airway (this phenomenon primarily occurs in the smaller airways and further enhances gas mixing and hence exchange); 2) convective dispersion due to asymmetric velocity profiles, which describes the asymmetry between inspiratory and expiratory velocity profiles leading to fresh gas movement more distally in the lungs; 3) cardiogenic mixing, which is when the beating of the heart contributes to a movement of gas in the pericardial regions of the lungs that promotes gas mixing; 4) molecular diffusion, which is the movement of molecules from higher concentration to lower concentration within small spaces and across membranes, in this case the alveoli; 5) pendelluft, which describes the movement of gas between lung units with different time constants, a property related to the product of compliance and resistance (following inspiration, there is redistribution of inspired gas from full, fast-filling units to slower-filling units, augmenting gas exchange); and 6) bulk flow, which may contribute partially to gas exchange as the leading edge of the gas front may actually reach a number of proximal alveoli—solid evidence for this mode of action is still lacking.

## Currently available high-frequency jet ventilators for use during general anaesthesia

**Figure 1.**
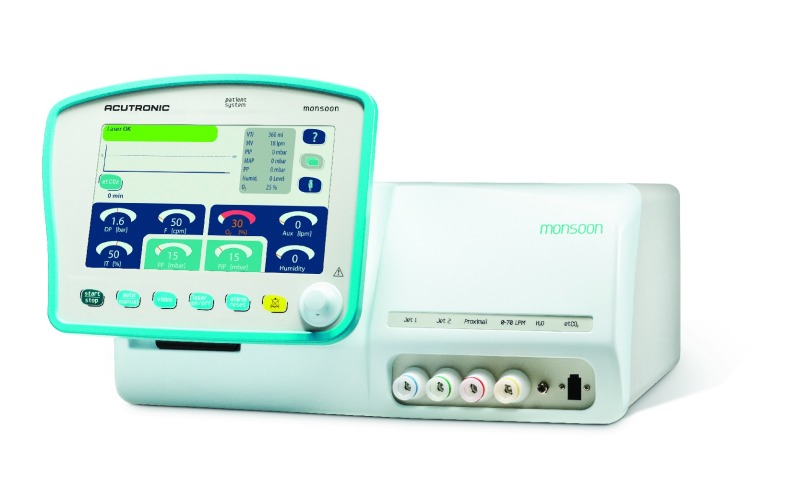
Monsoon jet ventilator. “Monsoon” ventilator (Acutronic Medical Systems, Ag, 8816 Hirzel, Schweiz).

**Figure 2.**
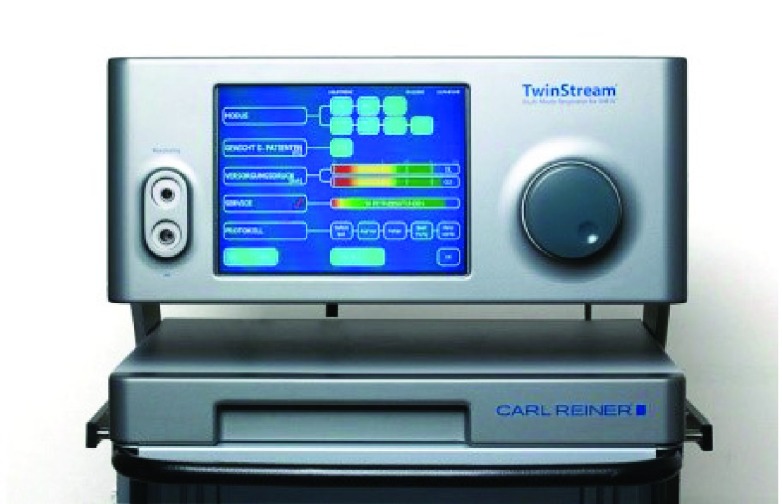
Twinstream. “Twinstream” ventilator (Carl Reiner GmbH, Vienna, Austria).

**Figure 3.**
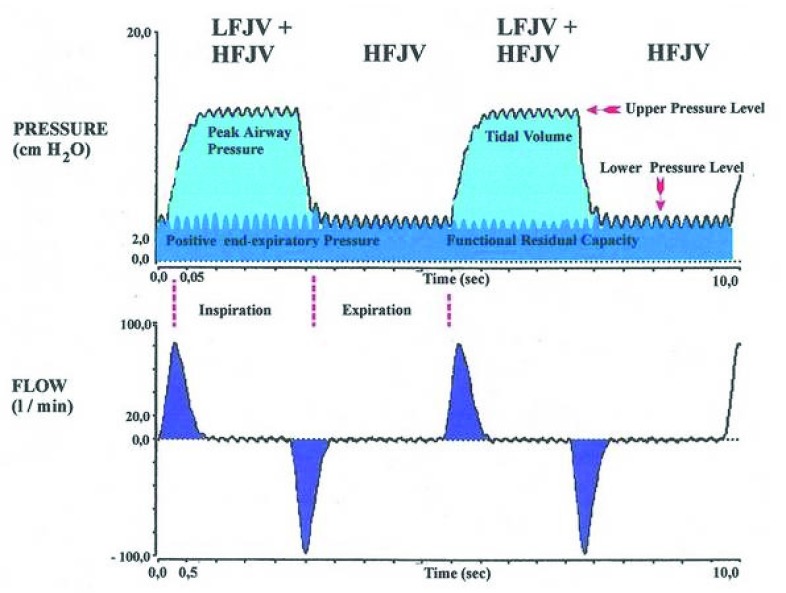
Illustration of superimposed high-frequency jet ventilation (HFJV). LFJV, low-frequency jet ventilation.

Two ventilators for jet ventilation during anaesthesia in non-neonatal care dominate the current market. These ventilators include “Monsoon” (Acutronic Medical Systems, Ag, 8816 Hirzel, Schweiz) ( Figure 1) and “Twinstream” (Carl Reiner GmbH, Vienna, Austria) ( Figure 2). Both ventilators deliver ventilator breaths across a wide range of frequencies (4–1,600 breaths/minute). They are also capable of delivering high- and low-frequency ventilation simultaneously, usually named superimposed HFJV ( Figure 3). Superimposed HFJV is more effective in gaseous exchange compared to conventional HFJV, especially for CO _2_ removal. Superimposed HFJV is beneficial in some ear, nose, and throat (ENT) surgeries or in patients with severe obstructive or restrictive lung disease. The technique is less useful in ablation procedures, as the superimposed low frequency provides tissue movements that interfere with the procedure. Jet ventilators have settings that need to be managed, as in conventional ventilators. The volume of each pulse is determined by the driving pressure (bar), frequency (minute ^-1^), and I:E ratio. Inspired O _2_ (%) level and the level of humidification also require operator control.

There are also manually driven jet ventilators, including Sanders jet ventilator or Manujet III (VBM Medizintechnik GmbH-Germany, Sulz a. N. Germany), which are mainly aimed at emergency transtracheal rescue ventilation ^10, 11^.

## Jet ventilation catheters

**Figure 4.**
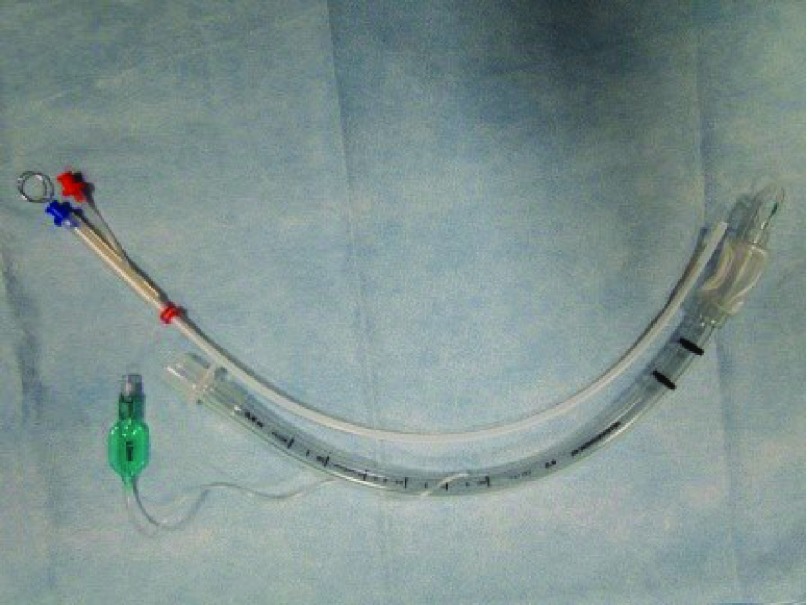
LaserJet and endotracheal tube. LaserJet Catheter (Acutronic Medical Systems AG, Hirzel, Switzerland).

No endotracheal tube is needed for supraglottic approaches. Several different devices are available for subglottic and transtracheal approaches. The Hunsaker Mon-Jet Catheter (Medtronic Xomed, Jacksonville, USA) or LaserJet Catheter (Acutronic Medical Systems AG, Hirzel, Switzerland) ( Figure 4) can be used for a subglottic approach. Both are double lumen cannulas with one lumen for gas delivery and one for gas and pressure monitoring. Both are non-flammable and made from laser-resistant material and have stylets to make insertion smooth. Many different devices for transtracheal jet ventilation exist, including Ravussins (VBM Medizintechnik GmbH-Germany, Sulz a. N. Germany), which has sizes for adults, children, and infants and can also be used in manually driven jet ventilators ^10^.

## Indications for use: why high-frequency ventilation?

Sjöstrand *et al*. initiated the use of HFV in ENT surgeries, and it has since then been used in different settings. The current applications of HFV are described below with a focus on HFJV.

### Ear, nose, and throat

HFJV is an attractive alternative ventilation technique when surgical field and airway management is in conflict. Diagnostic and/or therapeutic laryngoscopy demands that the vocal cords and the surrounding tissue are visible and easily available for interventions. Thus, standard tubes interfere and compete with the surgical field. HFV catheters are far less occupying and are commonly made of non-flammable material, thus reducing the risk of fire associated with the use of laser surgery ^10^.

### Thoracic surgery

HFJV by a catheter is also a valid alternative for bronchial procedures with or without the use of a double lumen tube. It lessens the conflict of space within the airways and increases visibility and surgical field. HFJV is also used successfully in the treatment of major broncho-pleural fistulae and tracheobronchial disruptions. Decreasing PAWP and tidal volumes result in smaller gas leaks through pathological low-resistance pathways. Consequently, mediastinal and interstitial emphysema may be minimised. This is today somewhat replaced by other techniques such as extracorporeal systems for oxygenation and removal of CO _2_, e.g. ECMO and ECCO2R.

### As part of the ventilation strategy in severe lung disease

This involves using HFV to provide ventilation and lung recruitment and to minimise airway and intrathoracic pressure, reducing right heart constraint and pulmonary artery pressures. HFV is used in patients with respiratory distress syndrome in the neonate as well as a rescue therapy in ARDS. The use of HFV in acute lung injury improves oxygenation, but its effects on clinical course, outcome, and survival are less clear ^12^.

### Minimising movements associated with normal tidal volume breathing

Another reason to use HFV is to facilitate minimising surgical field motion. Froese and Bryan described the use of HFV for the laser treatment of lung tumours ^4^. Some recently published reports describe the use of HFJV in other fields such as interventional radiology and non-ENT surgery, where the inhibition of respiratory movement is important for procedural precision. A few examples are minimally invasive treatment of tumours with different techniques and thermal ablation and irreversible electroporation (IRE), including percutaneous, laparoscopic, and open approaches ^13– 16^. HFJV is beneficial in cardiology when used alongside interventions such as catheter ablations for atrial fibrillation ^17^. In urology, HFJV can be used in extracorporeal shock wave lithotripsy (ESWL) surgery to minimise the number of shocks needed ^18^. HFJV is also used as a rescue method in the treatment of a difficult airway, as described by the pioneers.

Thermal ablation of tumours in solid organs is one of the most promising and dynamically developing procedures in clinical oncology. A wide range of devices and techniques are entering the clinical setting, offering a new approach in the treatment of both primary and secondary malignancies in a much less invasive way compared with conventional surgery. Targeting anatomically difficult located tumours is driving innovation in radiological imaging as well as the implementation of stereotactic techniques in this field. High precision in surgical manipulation becomes even more dependent on immobilisation of the operation field when malignant lesions are targeted with the use of image fusion with previous diagnostic imaging ^13, 15^. There is a need for ventilator techniques that minimise movement while maintaining a near-static state of the ablation target area.

**Figure 5.**
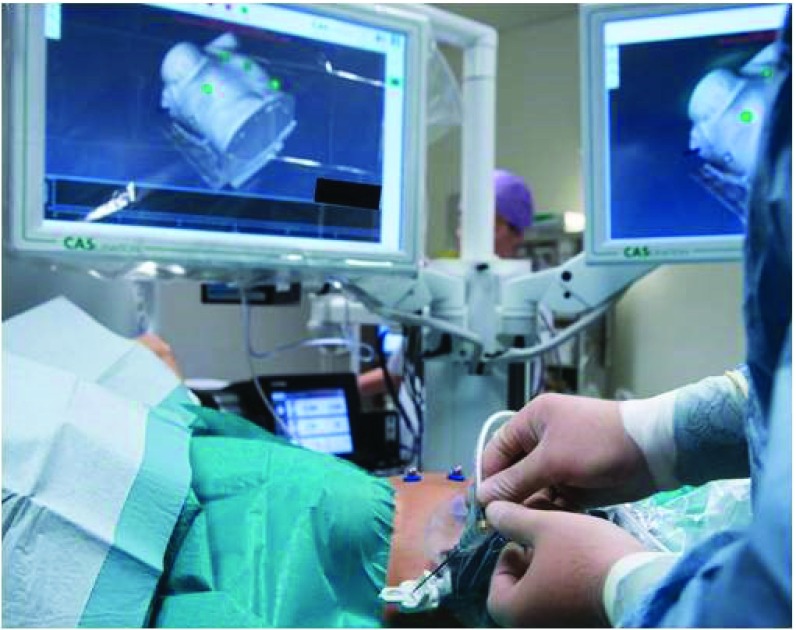
Liver tumour ablation with high-frequency jet ventilation. Liver tumour ablation with high-frequency jet ventilation.

**Figure 6.**
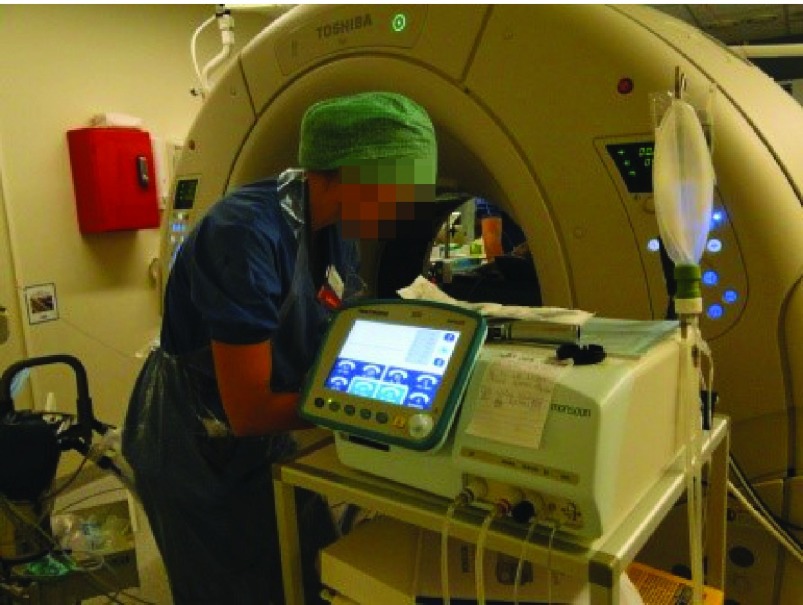
High-frequency jet ventilation used in the computed tomography lab during liver tumour ablation. High-frequency jet ventilation used in the computed tomography lab during liver tumour ablation.

Respiratory movements influence organs in the vicinity, causing respiratory movement, thus there could be dislocation during a stereotactic-guided procedure ( Figure 5 and Figure 6). Biro *et al*. observed that breathing-related liver motion decreased from 20 mm to 5 mm using HFJV compared to conventional ventilation ^15^. HFJV is an attractive ventilator strategy for minimising the movement of tissues in close proximity to the lung, keeping the abdominal and extraperitoneal target organ immobilised.

## Problems and drawbacks of high-frequency jet ventilation

HFJV is an open system where expiration is passive. As it is open to the atmosphere, total intravenous anaesthesia including muscle relaxation is the preferred anaesthetic technique. What is worth mentioning is that it is not possible to measure end-tidal CO _2_ (ETCO _2_) continuously with a system open to the atmosphere. This problem can be solved by pausing the HFJV regularly, so that the jet ventilator samples gas during a few deeper breaths for a more accurate measure of ETCO _2_. This function comes as a standard in at least some of the devices. Our experience is that this ETCO _2_ measurement differs appreciably, commonly giving false low values, from PaCO _2_, especially as the gas in some situations, such as laryngoscopy, is sampled above the vocal cords and probably does not reflect the CO _2_ content within the distal airways. Another method is the use of transcutaneous CO _2_ (TcCO _2_) measurement. The accuracy of this type of monitoring is independent of pulmonary status and may bypass some of the problems inherent to ETCO _2_ monitoring.

As expiration is passive, there is a need for enough time for air to be “exhaled/passed out into the surrounding atmosphere”. The higher the frequency, the less time for expiration; this could in turn lead to a build-up of an intrinsic PEEP ^19, 20^. One feared complication of HFJV is the potential for a build-up of an occult PEEP, leading to high airway pressures and dynamic hyper-insufflation and subsequently barotrauma, e.g. pneumothorax. Accidental high intrathoracic pressure may also possibly compromise venous return and increase intracranial pressure. Patients with a history of severe obstructive lung disease, severe cardiovascular compromise, and elevated ICP must thus be acknowledged. The explicit risk associated with HFJV as compared to traditional positive pressure ventilation has been shown to be relatively minor. In a study with only 10 patients but with severe obstructive lung disease, no difference was found regarding efficiency of ventilation (PaCO _2_), haemodynamic effects (stroke volume, blood pressure, and cardiac output), or lung hyperinflation (trapped gas volume) when HFJV was compared to “optimal” IPPV ^21^. Current HFJV ventilators monitor airway pressure and abort ventilation and start an alarm signal if the airway pressures reach a predetermined level. Alarms for pressure control given by the jet ventilator (peak inspiratory pressure, pause pressure, and MAWP) are also essential: pressure limits must be set prior to anaesthesia where the jet ventilator aborts ventilation and gives an alarm signal to avoid barotrauma.

Humidification is essential during HFJV, and if this is impaired there is a risk for tracheal mucosa dehydration and subsequent necrosis. There are built-in humidification systems in modern HFJVs ^22, 23^.

## Discussion

HFV is not a new ventilation method. Its place in the intensive care unit is a matter of debate. Recent meta-analysis has not shown convincing effects of HFOV on clinical outcome in ARDS ^12^. The use of HFV during general anaesthesia has been uncommon over recent decades. However, there is a renewed interest in the use of HFJV during general anaesthesia. Many surgical procedures can benefit from less breathing-related movement, giving the surgeon better working conditions and, more importantly, improving the performance of stereotactic procedures in the liver and other organs in close proximity to the lung/diaphragm.

HFJV is an unconventional technique with a steep learning curve and thus it demands being used by trained personnel following a proper assessment of benefit versus risk and with appropriate procedures for monitoring. Basic monitoring such as SpO _2_, ECG, and blood pressure are essential during HFJV. Risks due to hypoventilation (and hyperventilation) must be acknowledged (see above), and CO _2_ monitoring is essential, irrespective of adequate oxygen saturation. CO _2_ monitoring is achieved by intermittent measure of ETCO _2_, TcCO _2_, or CO _2_ via arterial blood gases (PaCO _2_). TcCO _2_ monitoring provides an accurate estimation of PaCO _2_ in the HFOV-treated paediatric population ^24^. However, the slow response time for the cutaneous measurement must be kept in mind.

## Conclusion

There is an increasing interest in minimising movement secondary to ventilation, so-called motion management, for different ablation techniques. HFV seems to be an important potential ventilator strategy in order to improve performance and shorten procedure time. There is, however, a need for further studies verifying its benefits and risks in this setting.

## Abbreviations

ARDS, acute respiratory distress syndrome; ETCO _2_, end-tidal CO _2_; HFV, high-frequency ventilation; HFJV, high-frequency jet ventilation; HFOV, high-frequency oscillatory ventilation; HFPPV, high-frequency positive pressure ventilation; MAWP, mean airway pressure; PaCO _2_, partial pressure of Co2; PAWP, peak airway pressure; PEEP, positive end-expiratory pressure, TcCO _2_, transcutaneous CO _2_.
